# DsiRNA‐mediated silencing of *Ceratitis capitata transformer* or *transformer‐2* leads to masculinization of XX embryos and systemic gene silencing in ovaries

**DOI:** 10.1111/1744-7917.70173

**Published:** 2025-10-05

**Authors:** Gennaro Volpe, Sarah Maria Mazzucchiello, Domenico De Falco, David Torrente, Stefania Liguori, Noemi Rosati, Dora Baccaro, Michela Mazzeo, Fulvio Bertolotto, Harshini Sangle, Ennio Giordano, Angela Carfora, Francesca Lucibelli, Marianna Varone, Paola Di Lillo, Hugo Diego Perdomo, Mariangela Bonizzoni, Serena Aceto, Marco Salvemini, Molly Duman‐Scheel, Giuseppe Saccone

**Affiliations:** ^1^ Department of Biology University of Naples Federico II Naples Italy; ^2^ Department of Genetics, Microbiology, and Statistics, Faculty of Biology University of Barcelona Barcelona Spain; ^3^ Department of Agricultural Sciences University of Naples Federico II Naples Italy; ^4^ Department of Biology and Biotechnology University of Pavia Pavia Italy; ^5^ Department of Medical and Molecular Genetics Indiana University School of Medicine South Bend Indiana USA

**Keywords:** DsiRNAs, Mediterranean fruit fly, sex determination, sexing, Sterile Insect Technique, systemic RNAi

## Abstract

*Ceratitis capitata* (medfly), a major agricultural pest, is predominantly controlled using chemical insecticides, which pose environmental risks. Ecosustainable alternatives, such as the Sterile Insect Technique (SIT), rely on the mass release of sterile male‐only progeny. However, sexing of male offspring requires the elimination of females during development. To overcome the loss of 50% of the progeny, the sex reversal of females into XX fertile males at embryonic stages by dsRNA injections was effective but not scalable. This study demonstrates the efficacy of Dicer‐substrate small interfering RNAs (DsiRNAs) as an alternative to long double‐stranded RNAs (dsRNAs) for targeting the sex determination genes *Cctransformer* and *Cctransformer‐2* in the medfly, which causes full masculinization of XX individuals. Injection of DsiRNAs into XX embryos induced the expression of male‐specific *Cctra* isoforms within a few hours, resulting in the development of adult masculinized XX flies. Additionally, thoracic injection of DsiRNAs in adult females achieved systemic gene silencing, reducing *Cctra/Cctra‐2* transcript levels in the ovaries by 75%–80% within 48 h. The reduced size of DsiRNAs compared to dsRNAs enhances their potential for alternative delivery methods, including embryo permeabilization, electroporation, and feeding in larvae or adult females. These findings provide a potential foundation for future scalable conditional masculinization of XX individuals rather than relying on female lethality, doubling male‐only productivity. Developing novel sexing methods as an alternative to transgenic approaches will expand the applicability of SIT.

## Graphical abstract

We carried out functional studies using Dicer‐substrate small interfering RNAs (DsiRNAs), targeting the sex determination genes *Cctransformer* (*Cctra*) and *Cctransformer‐2* (*Cctra‐2*) in the Mediterranean fruit fly (*Ceratitis capitata*). Microinjection of DsiRNAs into XX embryos (0‐1 h old) rapidly triggered the expression of male‐specific *Cctra* isoforms (during embryogenesis) leading to the development of fully masculinized XX adults. Thoracic injection of DsiRNAs mix (*Cctra/Cctra‐2*) into females, resulting in significant reduction of transcript levels of targeted genes in the ovaries and carcasses within 48 h.

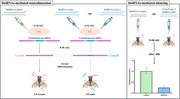

## Introduction

*Cctransformer* (*Cctra*), an orthologue of the *Drosophila melanogaster transformer* gene (Pane *et al.*, 2002), is a master gene for *Ceratitis capitata* female sex determination. In XX individuals, it produces female‐specific *Cctra* mRNAs, which encode a full‐length arginine‐serine‐rich (SR) CcTRA splicing factor (409 amino acid [aa]), and in XY individuals it generates male‐specific non‐functional mRNAs encoding CcTRAM1 (59 aa; GenBank: AAM88674.1) and M2 (99 aa; GenBank: AAM88675.1) truncated isoforms (Pane *et al.*, 2002). Similar to *D. melanogaster*, CcTRA interacts with CcTRA‐2, likely modulating female‐specific splicing of target pre‐mRNAs (Saccone *et al.*, 2008; Saccone, 2022; Perrotta *et al.*, 2023). However, differing from *D. melanogaster*, *Cctra* maintains female‐specific *Cctra* splicing through a positive feedback loop determining female sex (Pane *et al.*, 2002). Starting in early embryogenesis, female‐specific CcTRA and the non‐sex‐specific CcTRA‐2 serine/arginine‐rich SR splicing factors interact to bind *Cctra*, for its positive novel autoregulation, and *Ccdoublesex* (*Ccdsx*) pre‐mRNA regulatory elements, which were previously discovered in the *D. melanogaster dsx* orthologue (Tra/Tra‐2 binding sites, also known as *dsx*RE; Burtis & Baker, 1989). Transient embryonic repression of the *Cctra* and *Cctransformer‐2* (*Cctra‐2*) genes by injecting long dsRNA molecules (500–900 bp) led to the development of XX fertile males that were phenotypically indistinguishable from XY normal males. These experiments showed that medfly embryos are both very sensitive to transient embryonic RNAi and required for female sex determination (Pane *et al.*, 2002; Salvemini *et al.*, 2009). Similar to wild‐type XY male flies, the XX adult fertile males produced male‐specific *Cctra* mRNAs that encode for a non‐functional peptide, indicating that a stable shift in the *Cctra* sex‐specific splicing pattern induced by transient RNAi and the collapse of the positive autoregulation had occurred (Pane *et al.*, 2002).

The complete masculinization of XX individuals is particularly interesting because of its potential future applications in medfly control, leading to patenting of the method (Delli Bovi *et al.*, 2004). Developing novel sexing strategies, such as conditional male‐only progeny production to be integrated into the Sterile Insect Technique (SIT) is a primary goal in insect pest control. Currently, sexing is based on the use of the *C. capitata* VIENNA 8 strain (genetic sexing strain; Franz *et al.*, 2021). This strain carries two recessive mutations and a reciprocal Y‐autosome translocation, which allow for the selection of XY embryos by heat treatment and, at later stages, a second selection of brown pupae from white pupae by visual/automated selection. The XX individuals are sensitive to heat treatment (*tsl*) and show a white pupae (*wp*) phenotype. In contrast, XY individuals carry translocated wild‐type alleles of both autosomal genes linked to the Y chromosome, which also carries the *MoY* male‐determining gene (Meccariello *et al.*, 2019). VIENNA 8 is based on a discovery (pers. comm. of Gerald Franz and Carlos Cáceres to G.S.) of a *temperature‐sensitive recessive lethal* mutation that is tightly linked to a previously studied *white pupae* recessive mutation. A gamma‐ray induced reciprocal translocation of the autosomal region containing the corresponding wild‐type alleles and the Y chromosome led to build the VIENNA 8 strain (Kerremans & Franz, 1994). Furthermore, the double selection of XY embryos (resistant to heat treatment during embryogenesis and displaying a brown color at the pupal stage) rendered the VIENNA 8 strain robust and practical for large‐scale applications (Franz *et al.*, 2021). However, due to the unlikely discovery of two parallel mutations in two tightly linked genes, the transferability of this methodology to other species has proven difficult. Novel sexing approaches and gene‐editing strategies in tephritids, including the identification and modification of *wp* and *tsl* genes, have been developed (Meccariello *et al.*, 2017; Ward *et al.*, 2021; Sollazzo *et al.*, 2022; Sollazzo *et al.*, 2024; Aumann *et al.*, 2025). Various other biotechnological approaches, including tetracycline‐controlled female lethality or sex‐specific fluorescence sorting, were applied in *C. capitata* to select male‐only progeny for SIT (Fu *et al.*, 2007; Davydova *et al.*, 2023).

Alternatively, the ability to conditionally produce male‐only progeny through RNAi‐induced sexual transformation of XX individuals, rather than eliminating them via lethality or sex sorting, could potentially double productivity in mass‐rearing facilities and could potentially be used in transgenic development of novel gene drive approaches (Meccariello *et al.*, 2024). However, alternatives to transgenic approaches are needed given the regulations developed in many countries. Unfortunately, individual embryonic injections of dsRNA molecules are not scalable at an industrial level, and alternatives for a scalable delivery system are needed (Whyard *et al.*, 2015; Darrington *et al.*, 2017). DsRNA molecules are typically 200–500 bp long and difficult to deliver by embryo/larvae soaking methods. Much smaller molecules such as siRNAs (small interfering RNAs) or shRNAs (short hairpin RNAs), which can be delivered with alternative strategies, may prove useful. These smaller interfering RNAs are also effective for inducing RNAi, as shown in mammalian transfected cells (Elbashir *et al.*, 2001; Yu *et al.*, 2003) and later in lepidopteran‐ and dipteran‐injected embryos, in which mRNA reductions up to 90% were observed (Clemons *et al.*, 2011; Yamaguchi *et al.*, 2011; Flynt, 2021).

RNAi has also been induced at insect larval or adult stages by feeding dsRNAs, siRNAs, and shRNAs (Vogel *et al.*, 2019; Wiltshire & Duman‐Scheel, 2020; Mehlhorn *et al.*, 2021). Recently, in *C. capitata*, 3 days of co‐feedings in adult medflies with dsRNAs targeting the *v‐ATPase* and two intestinal *dsRNases* were effective in inducing 75% *v‐ATPase* mRNA reduction and 79% lethality within a week, confirming promising orally mediated RNAi in this species (Volpe *et al.*, 2024). Furthermore, medfly adult thorax injections and adult oral delivery of circular dsRNAs induced a *v‐ATPase* mRNA reduction of 90% and 48% after 3 days, respectively (Ortolá *et al.*, 2024). Interestingly, Ortolá *et al.* (2024) observed a substantial reduction in fertility and fecundity in dsRNA‐injected female adult medflies, which were suggestive of a systemic response. Similarly, in another tephritid species, *Bactrocera dorsalis*, dsRNA feeding induced strong RNAi silencing (mRNA reduction up to 80%–90%), which could be observed not only in the midgut, but also in other tissues like the ovaries, nervous system, and fat body, suggesting that the feedings had resulted in a systemic silencing effect (Li *et al.*, 2011).

As shRNA is a substrate for Dicer, it is more potent than its sequence‐matched siRNA (Siolas *et al.*, 2005). The yeast *Saccharomyces cerevisiae* has been used to express shRNAs corresponding to insecticidal siRNAs, facilitating cost‐efficient interfering RNA production during yeast cultivation (Mysore *et al.*, 2021). Similarly, Dicer‐substrate siRNAs (DsiRNAs) are chemically synthesized 27‐nucleotide RNA duplexes with 2‐nucleotide 3' overhangs that mimic natural Dicer products optimized explicitly for processing by the Dicer enzyme, which enhances the potency and efficacy of RNAi, engaging this natural processing pathway (Kim *et al.*, 2005; Amarzguioui *et al.*, 2006; Snead *et al.*, 2013). DsiRNA injection into adult female *Culex pipiens* mosquitoes during the early phase of diapause reduced mRNA levels of three genes by 70% after 7 days, revealing their stable and long‐term effects (Olademehin *et al.*, 2020).

In this study, we report that DsiRNAs induce the permanent masculinization of XX *C. capitata* embryos through adulthood and exert a systemic effect when injected in the female thorax, inducing strong gene silencing in the ovaries.

## Materials and methods

### Insect rearing

The wild‐type *C. capitata* strain (from P. A. Mourikis, Benakeion Institute of Phytopathology, Athens, Greece) was maintained under controlled laboratory conditions at 25–26 °C, 60%–70% relative humidity, and a 12 : 12 h light–dark photoperiod. Adult flies were provided with a 3 : 1 mixture of sugar, yeast extract, and water. Following mating, females deposited eggs on a vertical mesh surface, which allowed the eggs to fall into trays containing distilled water. Collected embryos were transferred to Petri dishes containing a self‐made artificial larval diet (400 mL distilled water, 30 g sugar, 30 g yeast powder, 30 g paper, 10 mL of 2.5% cholesterol solution, 8 mL of 4% benzoic acid [pH ≈ 2.8], and 2 mL of 2% hydrochloric acid). Third instar jumping larvae left the diet medium to pupate in plastic boxes. Pupae were subsequently transferred to clean Petri dishes and held until adult emergence.

### Design of DsiRNAs targeting Cctra and Cctra‐2 genes

The *Cctra* (LOC101456163) and *Cctra‐2* (LOC101452698) genes, required in the female sex determination of *C. capitata* embryos, were selected as targets for RNAi‐mediated masculinization of XX individuals (Pane *et al.*, 2002; Salvemini *et al.*, 2009). DsiRNAs were designed using the Integrated DNA Technologies (IDT, Coralville, IA) online tool and verified for specificity using NCBI BLAST analysis, confirming no significant homology to other *C. capitata* genes beyond the intended targets. Three distinct DsiRNAs were used for *Cctra* and five for *Cctra‐2* (see Table S1). A scrambled negative control DsiRNA duplex was employed in control experiments (see Table S1).

### Embryonic and adult injections

We microinjected wild type embryos with dsRNA targeting *Cctra*, as previously described (Pane *et al.*, 2002). This produced fertile sex transformed XX males lacking a Y chromosome, selected from XY males by molecular karyotyping, as described in Meccariello *et al.* (2019). These males were then individually crossed to 3 wild type virgin females each, to obtain embryos harboring no Y chromosome. Medfly XX embryos were collected 45 min after oviposition, and the chorion was manually removed. Dechorionated embryos were aligned and dehydrated in a calcium chloride (CaCl_2_) solution for 6 min, then overlaid with a layer of Halocarbon Oil 700 (Sigma‐Aldrich, Darmstadt, Germany) to prevent desiccation. Microinjections were performed under a Leica DM IRB inverted phase‐contrast microscope using quartz needles (O.D. 1 mm) pulled with a P‐2000 micropipette puller (Sutter Instrument Co., Novato, CA) with the following specifications: Heat 750, Fil 5, Vel 70, Del 130, Pull 175. After loading the injection mix into the needle, it was mounted on a micromanipulator arm (TransferMan^®^ 4r, Eppendorf SE, Hamburg, Germany) and connected to a FemtoJet 4i (Eppendorf SE, Hamburg, Germany) microinjector for delivery. Each DsiRNA was microinjected at a final concentration of 20 *µ*mol/L in an average of 100 XX embryos. Twenty‐four hours after injection, a subset of 100 embryos was collected for RNA extraction and RT‐PCR analysis to evaluate the potential masculinization effect at the molecular level. The most efficient DsiRNA candidates were then microinjected into XX embryos (an average of 100 embryos for each DsiRNA, see Table 1) to evaluate the percentage of masculinization at the adult stage. Emerged adults were first phenotypically examined and then processed for RNA extraction and RT‐PCR analyses.

**Table 1:** Masculinization of the XX‐only progeny following embryonic DsiRNAs injection

DsiRNAs	Injected XX embryos	Pupae	Tot. Adults	Males	Females	Intersex	% of males	% of intersex
*Cctra* #1	129	16	15	6	0	9	40%	60%
*Cctra* #2	150	20	18	12	0	6	67%	33%
*Cctra* #3	183	23	21	15	0	6	71%	29%
*Cctra*‐2 #1	88	11	5	4	0	1	80%	20%
*Cctra*‐2 #2	101	16	10	8	0	2	80%	20%
*Cctra*‐2 #5	105	21	12	11	0	1	92%	8%
DsiRNA scrambled	100	42	36	0	36	0	0%	0%
Injection buffer	100	55	46	0	46	0	0%	0%

Note: *Cctra‐2* #5 induced the highest percentage of masculinization and the lowest percentage of intersex phenotypes among treated embryos.

To assess RNAi efficiency at the adult stage, a mixed solution of all effective DsiRNAs *Cctra*/*Cctra‐2* (final concentration of 20 *µ*mol/L each) was injected into the thorax of ten adult females (flies were anesthetized by placing them on ice for 4–5 min), as well as for a mix of scrambled DsiRNAs (control). Microinjections were performed using the Nanoject III™ system (Drummond Scientific Co., Broomall, PA, USA), employing borosilicate glass needles (O.D. 1 mm; Drummond Scientific Co., Broomall, PA, USA) pulled with a P‐1000 micropipette puller (Sutter Instrument Co., Novato, CA; Heat 563, Vel 120, Time 2.00, Pull 30) inserted into the thoracic region, just below the base of the wing. At 48 h post‐injection, five treated females from each biological group were dissected to separate the ovaries from the remaining carcass and were processed for RNA extraction and subsequently analyzed by quantitative PCR (qPCR) to assess the expression levels of *Cctra* and *Cctra‐2*. The remaining five injected females for each treatment were crossed with 15 XX males to assess the potential masculinization of the progeny.

### RNA extraction

Total RNA was isolated from pooled embryos and individual adult flies and tissues using TRIzol™ Reagent (Thermo Fisher Scientific, Waltham, MA, USA), following the manufacturer's protocol. RNA concentration and purity were assessed using a NanoDrop 2000c spectrophotometer (Thermo Fisher Scientific, Waltham, MA, USA). Genomic DNA contamination was eliminated by treating RNA samples with RNase‐free DNase I (NEB, Ipswich, MA, USA). The effectiveness of DNA removal was verified by RT‐PCR targeting the intron‐containing *Ccsod* gene (Primo *et al.*, 2020). Following RNA extraction and quantification, 1 *µ*g of total RNA was reverse transcribed using the LunaScript RT SuperMix Kit (NEB, Ipswich, MA, USA).

### RT‐PCR analysis

RT‐PCR expression analysis was performed on the cDNA of embryos and adult flies using One*Taq*® 2× Master Mix with Standard Buffer (NEB, Ipswich, MA, USA) with the primers reported in Table S2. The amplicons were visualized through gel electrophoresis, with a 100‐bp ladder (NEB, Ipswich, MA, USA).

### Real‐time quantitative PCR analysis

Total RNA from each biological replicate (5 replicates for each treated group), consisting of ovaries and carcasses of injected adult females, was diluted to a final concentration of 50 ng/*µ*L for qPCR analysis. Quantitative real‐time PCR was performed using the QuantStudio 5 Real‐Time PCR System (Applied Biosystems, Carlsbad, CA, USA) to assess the effects of DsiRNA treatments on *Cctra* and *Cctra‐2* gene expression. Gene‐specific primers were used (see Table S2), and the ribosomal protein gene *RpL19* served as an internal reference control (Sagri *et al.*, 2017).

Relative gene expression was determined using the Power SYBR™ Green RNA‐to‐CT™ 1‐Step Kit (Applied Biosystems, Carlsbad, CA, USA), following the manufacturer's protocol. Data were analyzed using the 2^−ΔΔCt^ method (Livak & Schmittgen, 2001; Pfaffl, 2001). To validate this approach, the ΔCt values—calculated as the difference between the Ct of the target gene and the Ct of reference gene—were plotted against a series of RNA dilutions (400, 200, 100, 50, and 25 ng). The resulting standard curves for each target/reference pair showed slope values below 0.1, indicating comparable amplification efficiencies between the primers.

### Statistical analysis

Data normality was assessed using the Shapiro–Wilk and Kolmogorov–Smirnov tests. Target gene expression levels were analyzed using a Student's *t*‐test. All statistical analyses were performed using GraphPad Prism version 9.0 (GraphPad Software, San Diego, CA, USA).

## Results

### Efficacy of DsiRNAs targeting Cctra and Cctra‐2 genes at the embryonic stage

**Fig. 1:**
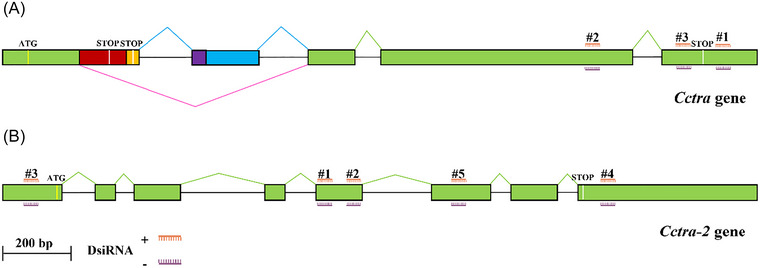
Genomic structures of *Cctra* (A) and *Cctra‐2* (B) and positions of DsiRNAs. Sense and antisense sequences are shown in orange and purple, respectively. Three DsiRNAs targeting the *Cctra* gene map to the third and fourth common exons (green boxes), while five DsiRNAs targeting the *Cctra‐2* gene map to the first, fifth, sixth, and eighth exons.

By crossing XX reverted fertile males (*Cctra*‐embryonic RNAi) with XX females, we produced female‐only XX embryos to more easily detect even rare masculinization events in the progeny (Pane *et al.*, 2002; Meccariello *et al.*, 2019). We injected 1‐h‐old female‐only XX embryos with DsiRNAs targeting the *Cctra* and *Cctra‐2* genes. We tested three DsiRNAs targeting the *Cctra* gene and five targeting the *Cctra‐2* gene (Fig. 1A, B; sequences in Table S1). These DsiRNAs were individually injected (20 *µ*mol/L) into 1‐h‐old female XX embryos to assess their potential to induce gene silencing and hence *Cctra* male‐specific splicing during embryogenesis of XX individuals. RNA was extracted from each pool of 100 injected XX embryos following 24‐h development for subsequent RT‐PCR analyses.

**Fig. 2:**
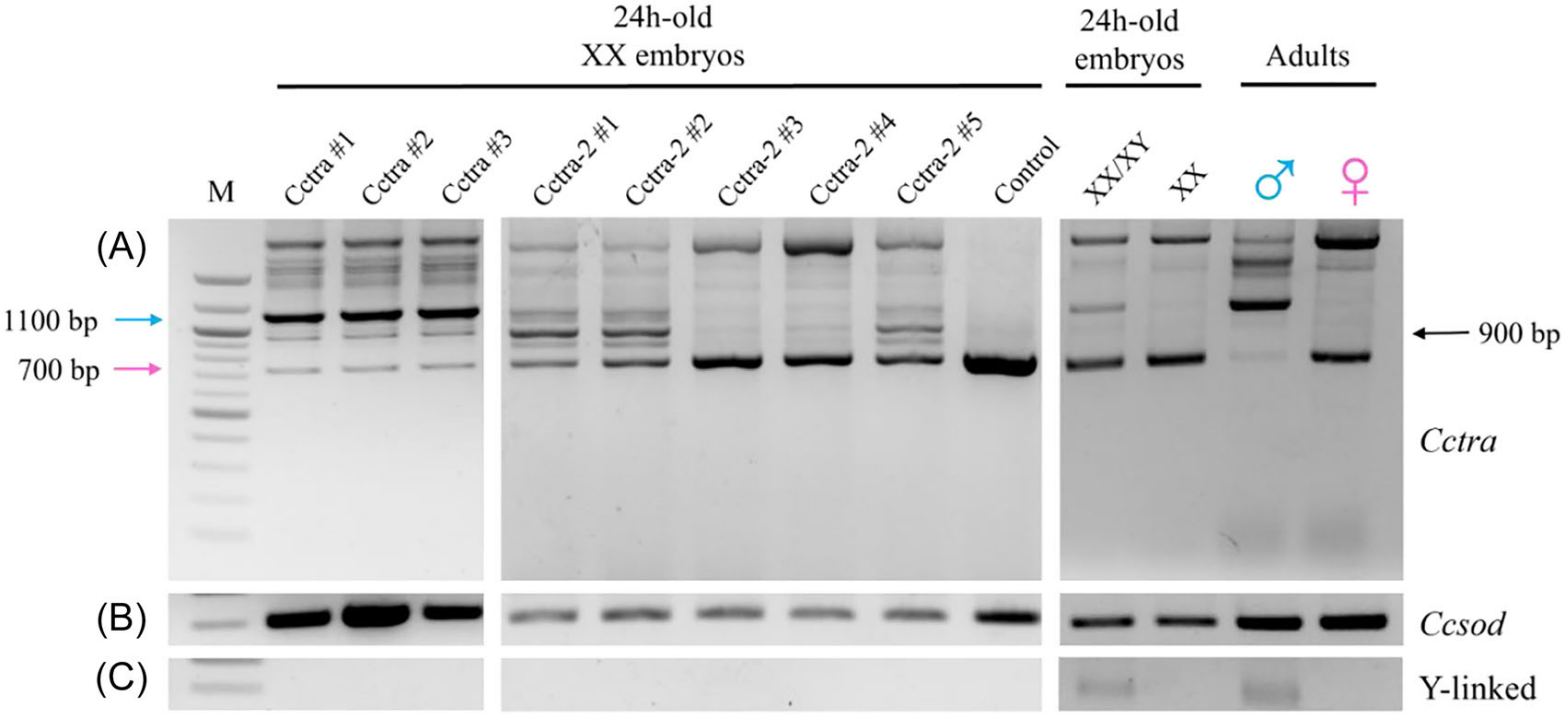
*Cctra* splicing pattern in injected XX 1‐h‐old embryos following 24 h of development. (A) RT‐PCR analyses showing the splicing pattern of *Cctra* mRNA: the blue arrow indicates the major male‐specific splice variant (∼1100 bp), the pink arrow indicates the major female‐specific variant (∼700 bp). Unspliced RNAs and intermediate splicing isoforms are also observed, as previously reported. The black arrow marks a new band (∼900 bp), which was subsequently sequenced and analyzed. Three adult flies of each sex were used for RT‐PCR of the last two lanes. (B) RT‐PCR analyses showing *Ccsod* mRNA expression (∼300 bp) were used as a positive control and a negative control for DNA contamination (no intron‐containing 400 bp band detected). (C) RT‐PCR analyses showing the presence/absence of a transcript derived from a Y‐linked embryonic gene (∼300 bp, *MoY*; Meccariello *et al.*, 2019) used as a negative and positive control for the molecular karyotyping. M: 100 bp DNA Ladder (NEB); Control: XX embryos injected with scrambled DsiRNAs.

All three *Cctra* #1, #2, and #3 DsiRNAs induced a splicing shift leading to 1.1 Kb long *Cctra* male‐specific band corresponding in length to the one observed in XX/XY embryos and in adult males (blue arrow) (Fig. 2A), indicating efficient targeting of this gene by all DsiRNAs. Three out of five tested *Cctra‐2* DsiRNAs (*Cctra‐2* #1, #2, and #5) induced a weaker *Cctra* male‐specific isoform, encoding the truncated, hence likely non‐functional, CcTRA M2 male‐specific isoform (Fig. 2A, blue arrow; Fig. S1, M2 isoform). Interestingly, we observed a more abundant, shorter and novel 0.9 Kb aberrant *Cctra* splicing isoform, likely encoding the above mentioned M2 isoform (Fig. 2A, black arrow; Fig. S1, M3 isoform [unknown]).

### Efficacy of DsiRNAs in inducing the masculinization of XX flies developed from treated embryos

The six out of eight DsiRNAs that were the most effective (Fig. 2) at inducing male‐specific *Cctra* splicing were individually injected into 1‐h‐old XX embryos to evaluate phenotypic effects after full development into adults. In the six experiments, all XX individuals who reached adulthood were either fully or partially masculinized, showing intersexual mosaic traits (Table 1). The survival rate of specific DsiRNA‐injected embryos ranged between 5% and 21%, and 36% for DsiRNA scrambled controls. This variability is likely due to differences in the sharpness of each injection needle and in the manual procedure executed by different operators. The *Cctra‐2* DsiRNAs seemed more efficient in inducing full masculinization (80%–92% masculinization) than *Cctra* DsiRNAs (40%–71% masculinization).

**Fig. 3:**
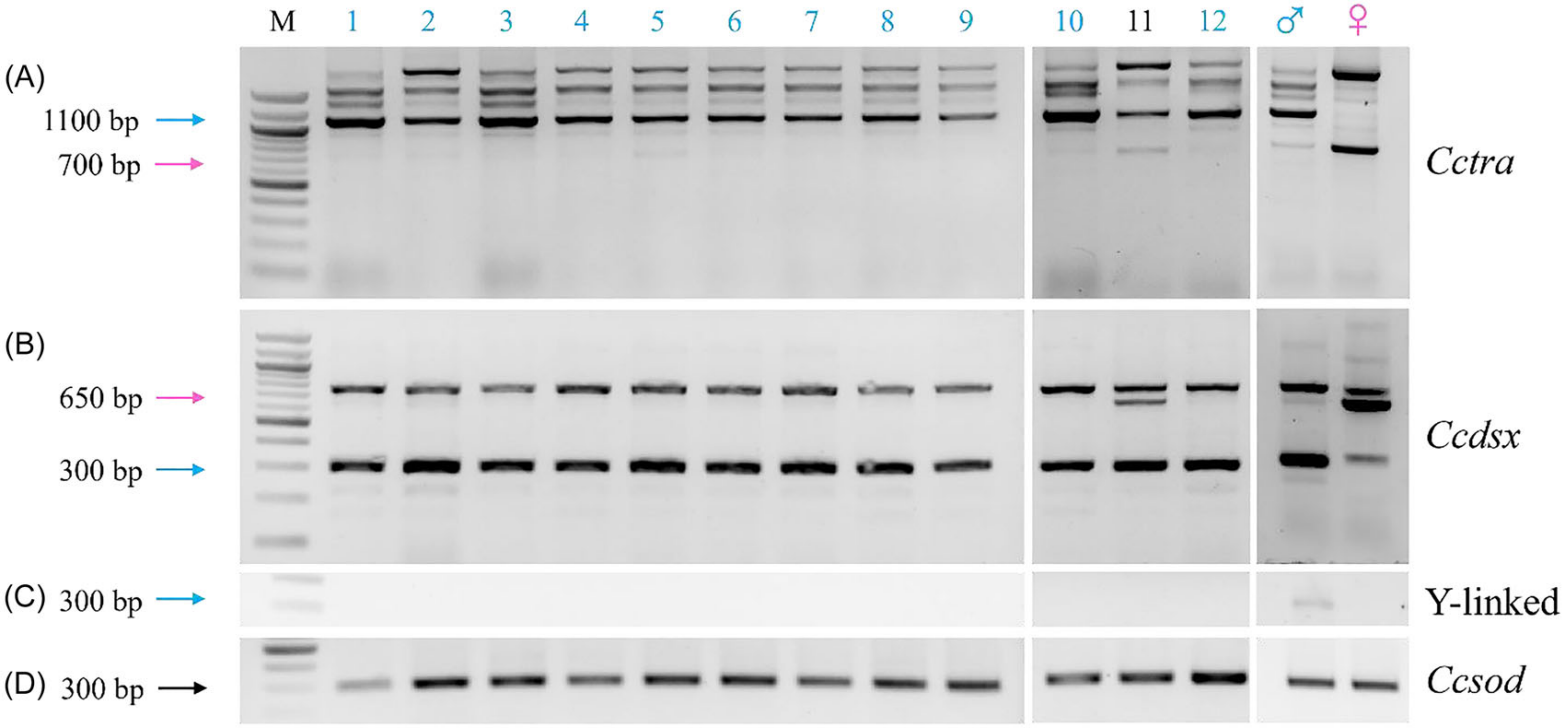
RT‐PCR of *Cctra* and *Ccdsx* in XX‐only adults developed from embryos injected with *Cctra‐2* #5. (A) RT‐PCR analyses showing the splicing pattern of *Cctra* mRNA: the blue arrow indicates the male‐specific splice variant (∼1100 bp), and the pink arrow indicates the female‐specific variant (∼700 bp). (B) RT‐PCR analyses showing the splicing pattern of *Ccdsx* mRNA: the blue arrow indicates the male‐specific splice variant (∼300 bp), and the pink arrow indicates the female‐specific variant (∼650 bp). (C) RT‐PCR analyses showing the presence/absence of a transcript derived from a Y‐linked embryonic gene (∼300 bp, *MoY*; Meccariello *et al.*, 2019) used as a negative and positive control for molecular karyotyping. (D) RT‐PCR analyses of *Ccsod* mRNA expression (∼300 bp) used as a positive control of cDNA and as a negative control for DNA contamination (no intron‐containing 400 bp band detected). Blue numbers indicate male individuals confirmed by both phenotypic and molecular analyses; black numbers indicate intersex individuals confirmed by the same criteria. M: 100 bp DNA Ladder (NEB).

RT‐PCR analyses of *Cctra* and *Ccdsx* (both regulated by *Cctra* and *Cctra‐2*) splicing patterns were performed in XX males and XX intersexes developed from embryos injected with *Cctra* or *Cctra‐2* DsiRNAs (Figs. 3 and S2–S6). They showed the presence of *Cctra* male‐specific mRNAs in most XX phenotypic males and of a mix of male‐ and female‐specific mRNAs in the intersexes, confirming that embryonic transient RNAi induced a stable splicing pattern shift in the regulation of both genes (Figs. 3 and S2–S6).

**Fig. 4:**
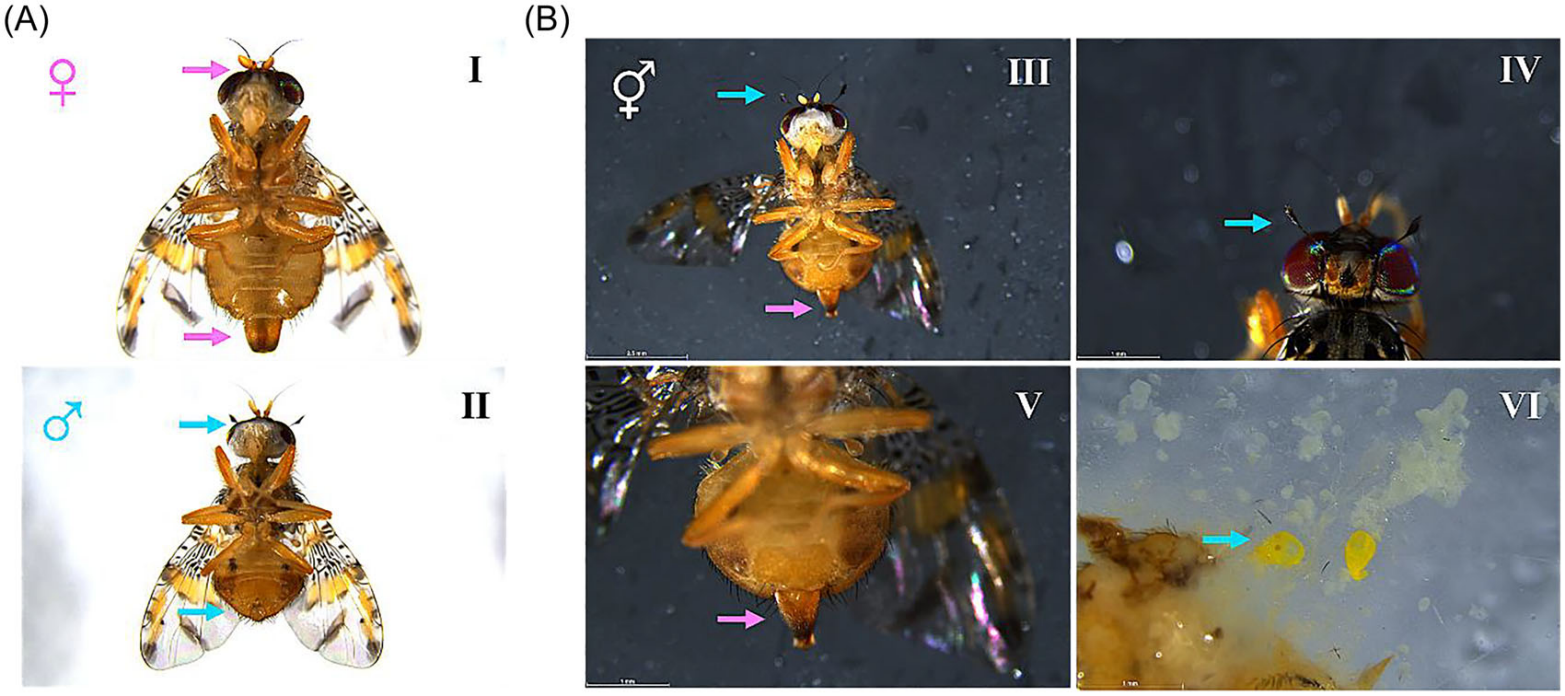
Phenotypic characterization of an intersex individual. (A) Representative images of wild‐type individuals displaying typical female (I) and male (II) morphological traits. (B) Example of an XX intersex individual (*Cctra‐2* #5 DsiRNA). Pink arrows highlight the female‐specific ovipositor in panels III and V; blue arrows indicate male‐specific antennae in panels III and IV and dissected testis in panel VI.

*Cctra* *‐2* #5 was the most effective DsiRNA, masculinizing 92% (11/12) of adult individuals (Figs. 1B and 3; Table 1). RT‐PCR analyses revealed exclusively male‐specific *Cctra* and *Ccdsx* mRNAs in 11 XX phenotypic males, suggesting complete masculinization at the molecular level. Furthermore, some individuals were mated with XX females and produced offspring (data not shown). On the contrary, this analysis showed a mix of male‐ and female‐specific mRNAs in the phenotypically intersexual XX fly (Figs. 3A, B and 4).

### Gene silencing efficacy of DsiRNAs injected into the adult female thorax

**Fig. 5:**
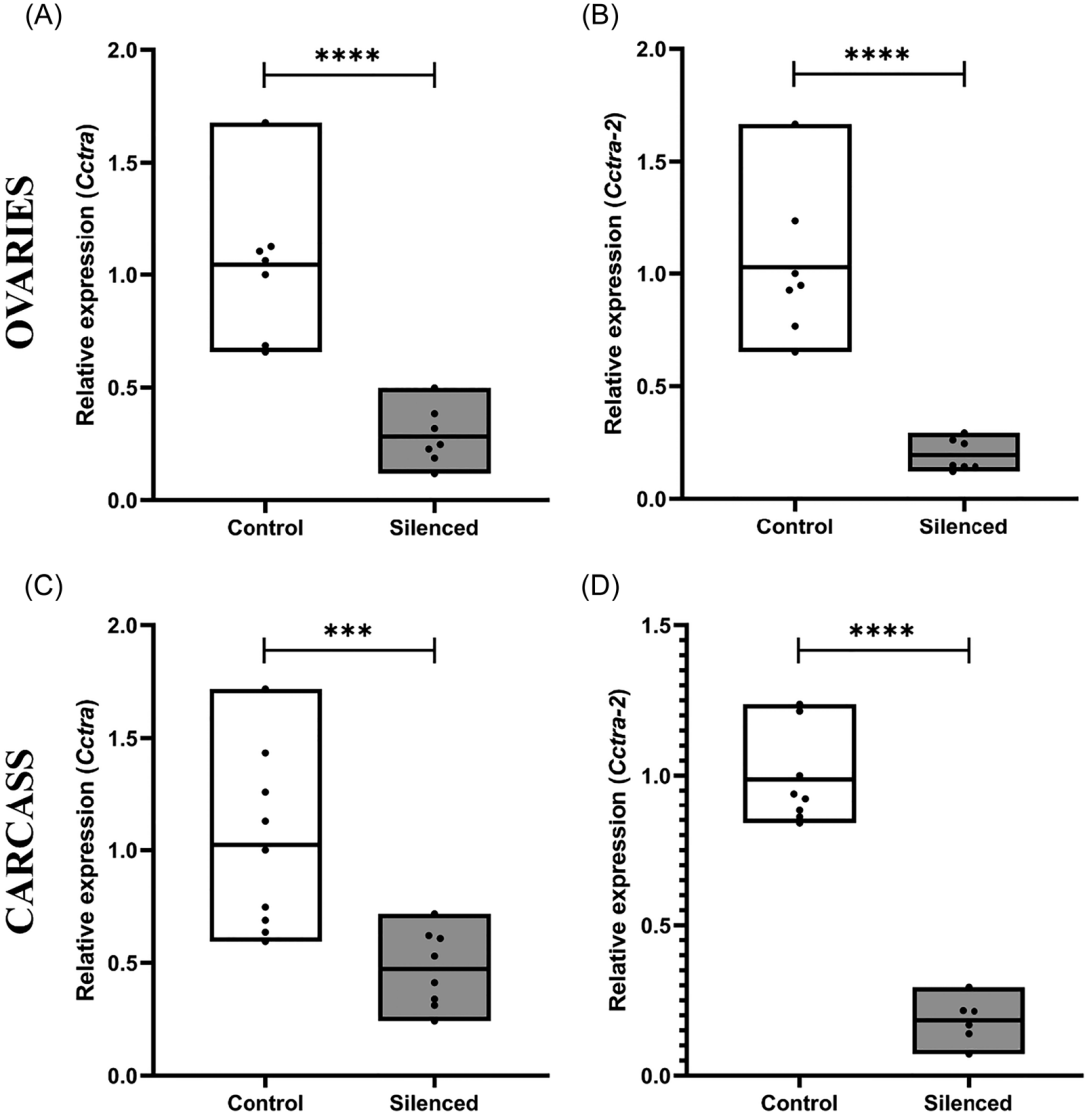
qPCR analysis of adult female ovaries and carcass following thoracic injection. (A) Relative transcript levels of the *Cctra* gene (Student's *t*‐test: *t* = 6.24, df = 12, ****P < 0.0001), and (B) relative transcript levels of the *Cctra‐2* gene (Student's *t*‐test: *t* = 9.433, df = 12, ****P < 0.0001) in the ovaries. (C) Relative transcript levels of the *Cctra* gene (Student's *t*‐test: *t* = 4.115, df = 15, ***P < 0.001), and (D) relative transcript levels of the *Cctra‐2* gene (Student's *t*‐test: *t* = 9.71, df = 12, ****P < 0.0001) in the carcass. The control group includes females injected with a scrambled DsiRNA mix, while the silenced group includes females injected with a *Cctra/Cctra‐2* DsiRNA mix. Asterisks indicate statistically significant differences between group means (all values passed normality tests). Data are presented as floating bars (min to max) + line at mean + individual data points.

We investigated whether DsiRNAs injected in adult females can diffuse into the ovaries, inducing efficient gene silencing. A mixture of *Cctra* and *Cctra‐2* DsiRNAs or a scrambled DsiRNA mix (for the control) were injected into the thorax of XX freshly emerged female flies. Two days post‐injection treated females were dissected to isolate the ovaries and the remaining carcasses, which were then processed for RNA extraction and subjected to qPCR analysis to evaluate the expression levels of *Cctra* and *Cctra‐2*. The quantitative analyses in the ovaries showed transcript level reductions, respectively, of 75% for *Cctra* and 80% for *Cctra‐2* (Student's *t*‐test: *P* < 0.0001) (Fig. 5A, B). Furthermore, qPCR analyses in the carcasses showed a reduction of 55% for *Cctra* and 80% for *Cctra‐2* (Student's *t*‐test: *P* < 0.001) (Fig. 5C, D). These results showed significant silencing of both target genes in the ovaries of adult females through the DsiRNA mix, suggesting a systemic delivery of small interfering molecules following adult thorax injections. However, five DsiRNAs‐injected XX females (3–4 days old when they reached sexual maturity) crossed with 15 XX young males produced a progeny of 150 individuals with no males or intersexes, indicating a lack of masculinization despite the observed reduction of *Cctra* and *Cctra‐2* mRNA levels in their ovaries.

## Discussion

Our study showed the capacity of DsiRNAs for reducing mRNA levels of *C. capitata* sex‐determination *tra* and *tra‐2* genes when delivered via injection at both the embryonic and adult stages. The DsiRNAs induce a stable *Cctra* splicing pattern change and masculinization when injected into embryos, leading to fully masculinized XX flies. Furthermore, when injected into adult female thorax, the DsiRNAs triggered strong gene silencing (75%–80%) in the ovaries. The stable shift in sex‐specific splicing patterns observed from embryonic stages through adulthood confirms that even transient interference of DsiRNAs with the *Cctra*/*Cctra‐2* autoregulatory loop during early development is sufficient to permanently redirect sexual differentiation (Pane *et al.*, 2002). This observation aligns with the current models of sex determination mechanisms in *C. capitata*, where the female‐specific splicing of *Cctra* must be established during embryogenesis to maintain female development (Pane *et al.*, 2002; Salvemini *et al.*, 2009; Primo *et al.*, 2020).

The masculinization percentage of XX individuals (11 out of 12 adult XX flies; up to 92% with *Cctra‐2* #5 DsiRNA) seems to be approximately comparable to results previously obtained using 500–900 bp long dsRNAs (Pane *et al.*, 2002; Salvemini *et al.*, 2009). However, the use of significantly smaller siRNA molecules (27 nucleotides) represents an important advancement for potential field applications, offering several advantages over conventional long dsRNAs. Their smaller size may facilitate delivery through alternative methods such as soaking, topical application, or oral administration, which can be scalable for a mass rearing facility (Darrington *et al.*, 2017; Wiltshire & Duman‐Scheel, 2020). They may also be less susceptible to degradation by insect nucleases, which pose a significant barrier to RNAi efficiency in many species (Volpe *et al.*, 2024). Although these molecules are potentially applicable in biotechnological contexts, it is necessary to test their efficacy using alternative delivery methods, determine appropriate concentrations to achieve near 100% masculinization rates, and develop synthesis systems that reduce production costs.

The detection of a novel non‐functional *Cctra* splicing mRNA isoform in all three *Cctra‐2* DsiRNA‐treated embryos, but not in *Cctra* DsiRNA‐treated embryos suggests a different mechanistic role of CcTRA‐2 in the binding of complex CcTRA/CcTRA‐2 to *Cctra* pre‐mRNAs. In *D. melanogaster* TRA‐2 binds specifically to the *tra/tra‐2* RNA cis elements (Hedley & Maniatis, 1991), and both TRA and TRA‐2 are found in a multiprotein complex assembled on this regulatory site *in vitro* (Tian & Maniatis, 1992; Tian & Maniatis, 1993). Though both *D. melanogaster* and *C. capitata* TRA‐2 display self‐interaction, the autoregulatory loop of *C. capitata* TRA is a distinction between the two insects (Perrotta *et al.*, 2023).

We observed variable efficacy among DsiRNAs targeting different sequences/regions of *Cctra* and *Cctra‐2* mRNAs, with those targeting *Cctra‐2* showing higher masculinization rates (80%–92%) compared to *Cctra* DsiRNAs (40%–71%). This discrepancy may reflect differences in gene sensitivity, and/or functional relevance of both genes, or the secondary structure and binding site accessibility of the targeted RNAs, or as well as intrinsic properties in the DsiRNA sequence (i.e., GC content, thermodynamic stability, asymmetry between 5' and 3' ends). DsiRNAs could not only induce RNAi in the cytoplasm but also interfere with the *Cctra* pre‐mRNA alternative splicing in the nucleus, physically hindering critical steps in splice site recognition or spliceosome assembly (Primo *et al.*, 2020). Novel siRNA efficacy predictive models based on machine learning are currently under development, promising faster improvement of their design (Tan *et al.*, 2025).

The significant reduction in *Cctra* (75%) and *Cctra‐2* (80%) transcript levels in ovaries following thoracic injection suggests that DsiRNAs can induce systemic silencing effects in adult flies, consistent with findings in other tephritid species such as *B. dorsalis* (Li *et al.*, 2011). The observed silencing in both ovaries and remaining carcass tissues suggests that *C. capitata* can uptake and spread small interfering RNAs quite efficiently despite lacking clear orthologs of the *C. elegans* systemic RNAi protein SID‐1 (as is common in dipterans). This systemic response is particularly notable given the ongoing debate regarding the efficiency of RNAi spread by dsRNA molecules in different insect orders (Gong *et al.*, 2015; Zhu & Palli, 2020). While robust systemic RNAi is well‐documented in coleopterans (Bucher *et al.*, 2002; Baum *et al.*, 2007), dipteran species often show more variable responses (Scott *et al.*, 2013). The robust silencing achieved in ovaries (75%–80%) is particularly significant from an applied perspective, as it suggests potential applications for transgenerational masculinizing effects for conditional male‐only progeny production or direct targeting of reproduction, as shown in *B. dorsalis* (Ali *et al.*, 2017). Nonetheless, such applications remain out of reach, as no masculinization was observed in the progeny of silenced females following thoracic injection, which in any case does not represent a suitable delivery method for large‐scale implementation.

While our study confirms the effectiveness of DsiRNAs in this species, future research should address certain limitations related to applied aspects to develop conditional masculinization strategies based on DsiRNA delivery during embryonic, larval, or adult stages. The first limitation to overcome is the delivery of DsiRNA into insect embryos at a large scale, which requires the development of novel permeabilization protocols and/or electroporation approaches for the medfly. Treatment with organic solvents such as hexane or isopropanol, which has been successfully applied in mosquito eggs (Chaverra‐Rodriguez *et al.*, 2018), could potentially be adapted for *C. capitata* to remove the waxy outer chorion layer and facilitate DsiRNA penetration. Combining permeabilization with osmotic pressure gradients could further enhance uptake efficiency across the vitelline membrane. Electroporation presents another viable strategy, as it has already successfully delivered long nucleic acids into insect embryos (DeVault *et al.*, 1996; Thomas, 2003; Ruiz *et al.*, 2015). The smaller size of DsiRNAs compared to plasmids or long dsRNAs should facilitate more efficient delivery via this method. Alternative delivery systems to the naked DsiRNAs, such as nanoparticle formulations, cell‐penetrating peptide conjugations should also be explored (Fernando *et al.*, 2022; Vogel *et al.*, 2023).

The second limitation is the lack of masculinizing effects of DsiRNAs delivered at adult stages in the ovaries. Surprisingly, when crossed with fertile XX males, the DsiRNA‐treated females, although showing strong *Cctra* and *Cctra‐2* mRNAs reduction (75%–80%) after 2 days from injections, continue to produce only female progeny. In contrast, an 80% reduction of *Cctra* mRNA alone in the medfly dissected ovaries by a transgene‐mediated maternal rather than embryonic RNAi led to masculinization of XX progeny (Volpe & Saccone, unpublished results). Although silencing was successfully achieved, maternal mRNA of *Cctra* and *Cctra‐2* is continuously deposited during the time window following our analyses and prior to oviposition, thereby negating the previously obtained effects. In contrast, the continuous deposition of dsRNA mediated by a transgene maintains low levels of *Cctra*, resulting in the masculinization of XX individuals. As oogenesis starts in late larval to early pupal stages, a technical solution to this problem would be the delivery of DsiRNA by continuously feeding larvae. For example, larval artificial feeding of chemically produced siRNAs successfully reduced transcripts of *acetylcholinesterase*, *coatomer β*, or *v‐ATPase A* in the lepidopteran cotton bollworm *Helicoverpa armigera*, decreased survival rate and body weight gain (Kumar *et al.*, 2009; Mao *et al.*, 2015). Interestingly, in this species, high levels of *acetylcholinesterase* silencing could be achieved using custom‐designed siRNAs, but not long dsRNAs (Kumar *et al.*, 2009). Another key challenge of this approach is to raise the percentage of masculinized XX individuals to at least 99.9%, as the observed 92% would not be sufficient for SIT application. The overcoming of present limitations in the embryo delivery and the DsiRNA‐efficiency in masculinizing will open the way for conditional DsiRNA‐induced sex reversal, rather than female elimination at a mass scale. This method could theoretically double the sexing efficiency of mass‐rearing operations by converting genetic females into phenotypic males rather than eliminating them with the available TSL system or transgenic strains.

A third limitation regards the cost of these molecules. Since their synthesis is not sustainable at an industrial level, it is necessary to develop alternative synthesis systems, possibly by employing heterologous expression vectors (e.g., yeast).

The following steps to advance this study will involve quantifying the DsiRNA‐induced reduction in mRNA levels in the ovaries at later time points following thoracic injections and after multiple thoracic injections. Additionally, mRNA levels will be assessed in both unfertilized and fertilized deposited eggs. Future research should also explore the potential for oral delivery of DsiRNAs, building on recent successes with dsRNA feeding in *C. capitata* as biopesticides (Ortolá *et al.*, 2024; Volpe *et al.*, 2024). RNAi‐based products for insect pest control are commercially available in the United States, with approvals from agencies such as the EPA. In contrast, the European Union regulates these products under frameworks overseen by the EFSA, treating them similarly to GMOs, and currently no RNAi‐based pest control products are widely commercialized.

The smaller size of DsiRNAs might facilitate intestinal absorption and systemic spread, particularly if protected from nuclease degradation through appropriate formulation or co‐delivery with nuclease inhibitors (Volpe *et al.*, 2024). Intra‐hemocoel injections of siRNAs complexed with cell‐penetrating peptides in the adults of the Orthoptera desert locust, *Schistocerca gregaria*, induced RNAi (Vogel *et al.*, 2023). Although siRNAs and shRNAs showed lower efficiency in triggering RNAi in the Colorado potato beetle *Leptinotarsa decemlineata* cells compared to long dsRNA, lipid‐mediated delivery improved their action (Koo *et al.*, 2023).

In conclusion, developing novel sexing methods as an alternative to transgenesis approaches could expand the applicability of SIT in countries resistant to the release of genetically modified insects, even when they are sterile.

## Disclosure

The authors have no conflict of interests to declare.

## Supporting information




**Fig. S1** Genomic organization and sex‐specific alternative splicing of the *Cctra* gene.
**Fig. S2** RT‐PCR of *Cctra* and *Ccdsx* in XX‐only adults developed from embryos injection with Cctra #1.
**Fig. S3** RT‐PCR of *Cctra* and *Ccdsx* in XX‐only adults developed from embryos injection with Cctra #2.
**Fig. S4** RT‐PCR of *Cctra* and *Ccdsx* in XX‐only adults developed from embryos injection with Cctra #3.
**Fig. S5** RT‐PCR of *Cctra* and *Ccdsx* in XX‐only adults developed from embryos injection with Cctra‐2 #1.
**Fig. S6** RT‐PCR of *Cctra* and *Ccdsx* in XX‐only adults developed from embryos injection with Cctra‐2 #2.
**Table S1** Sense and antisense sequences of designed DsiRNAs.
**Table S2** List of primers.
