# Supplementary material for: DsiRNA‐mediated silencing of Ceratitis capitata transformer or transformer‐2 leads to masculinization of XX embryos and systemic gene silencing in ovaries
Source: Insect Sci. 2025 Oct 5;33(2):586–98. doi: 10.1111/1744-7917.70173 (PMC13087853; doi:10.1111/1744-7917.70173)
Supplement: Supplementary file 1 — Fig. S1 Genomic organization and sex‐specific alternative splicing of the Cctra gene. Fig. S2 RT‐PCR of Cctra and Ccdsx in XX‐only adults developed from embryos injection with Cctra #1. Fig. S3 RT‐PCR of Cctra and Ccdsx in XX‐only adults developed from embryos injection with Cctra #2. Fig. S4 RT‐PCR of Cctra and Ccdsx in XX‐only adults developed from embryos injection with Cctra #3. Fig. S5 RT‐PCR of Cctra and Ccdsx in XX‐only adults developed from embryos injection with Cctra‐2 #1. Fig. S6 RT‐PCR of Cctra and Ccdsx in XX‐only adults developed from embryos injection with Cctra‐2 #2. Table S1 Sense and antisense sequences of designed DsiRNAs. Table S2 List of primers. [file INS-33-586-s001.docx]

**Supp. Data**

**
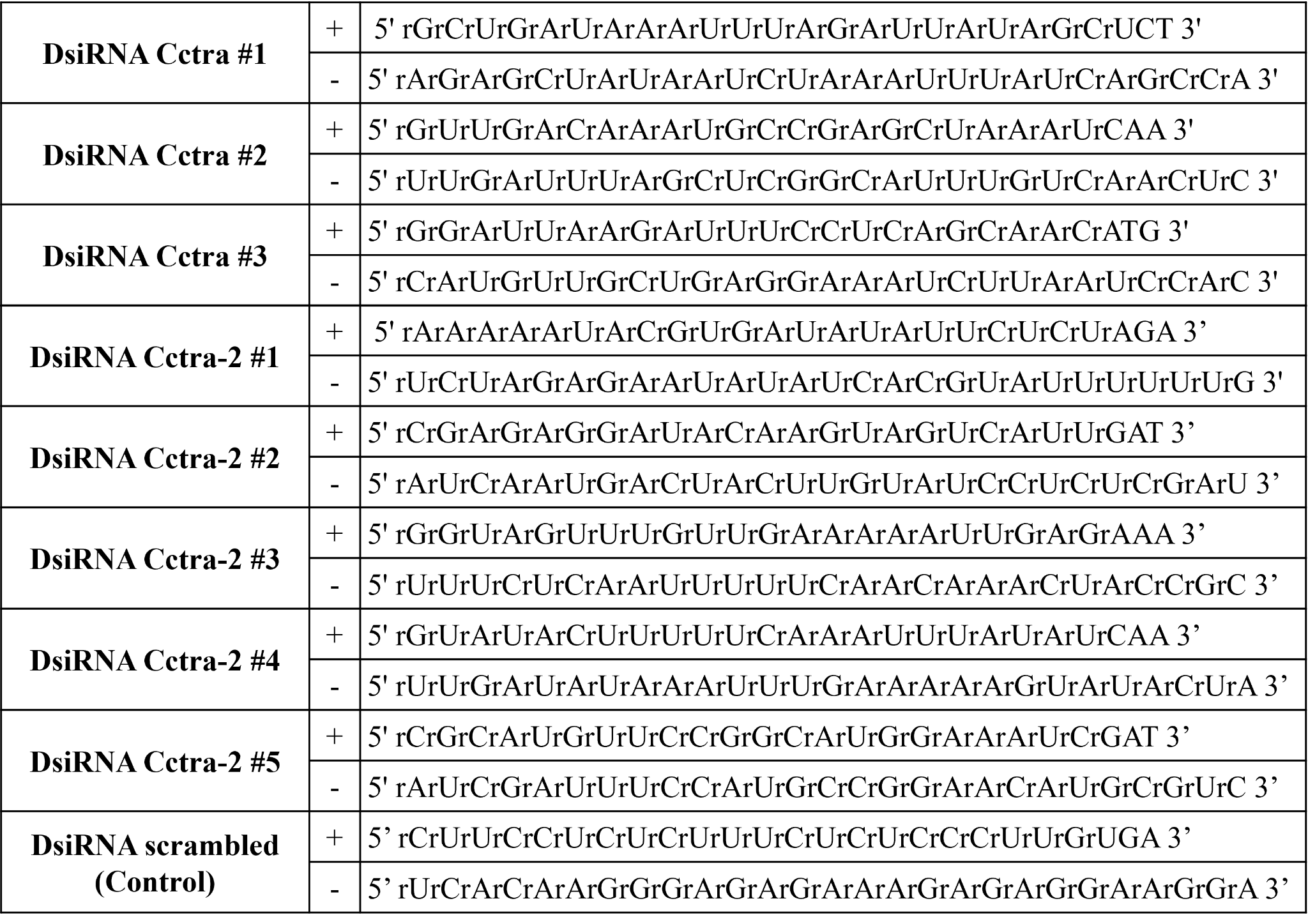
**

**Table S1. Sense and antisense sequences of designed DsiRNAs.**


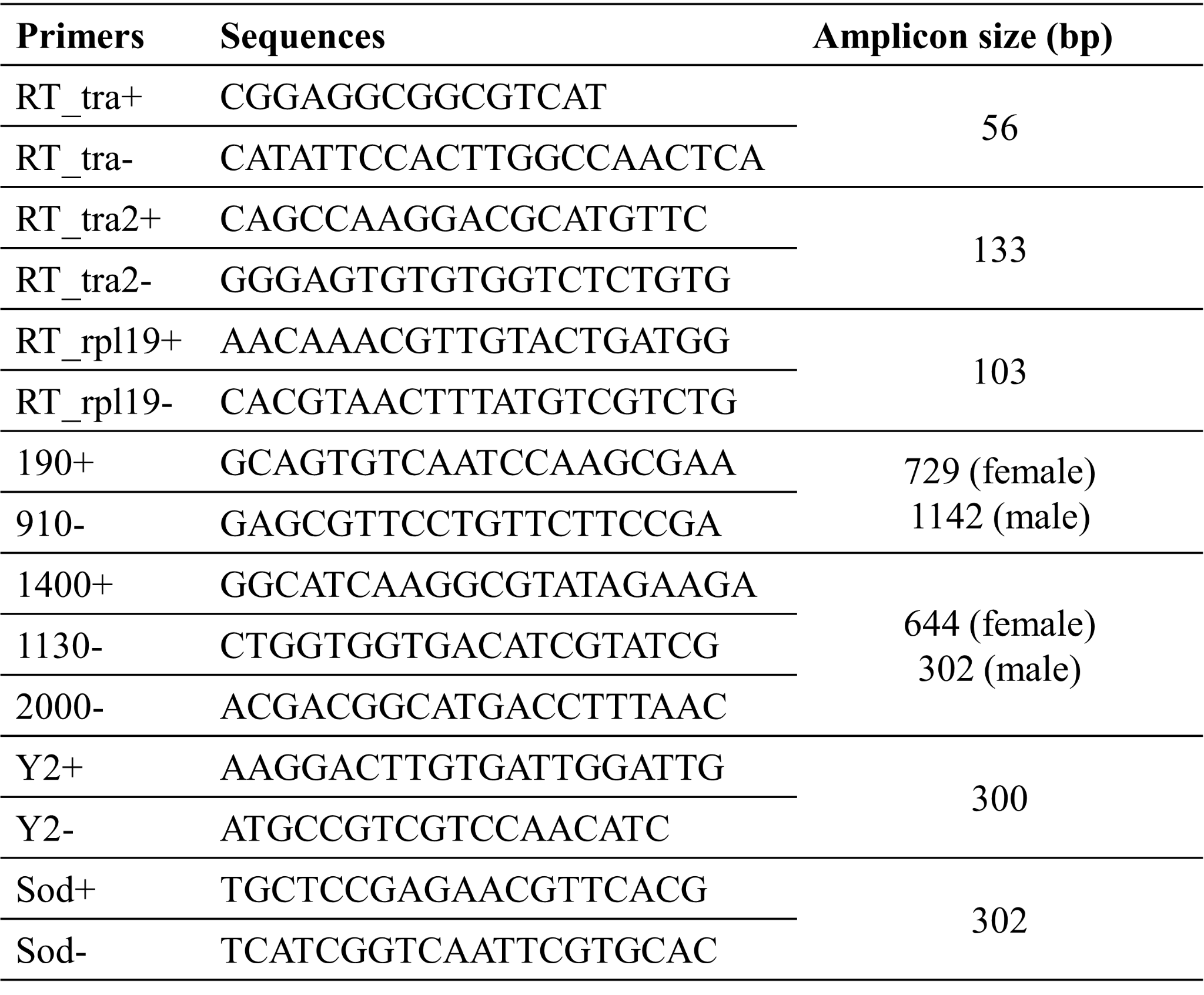


**Table S2. List of primers.**


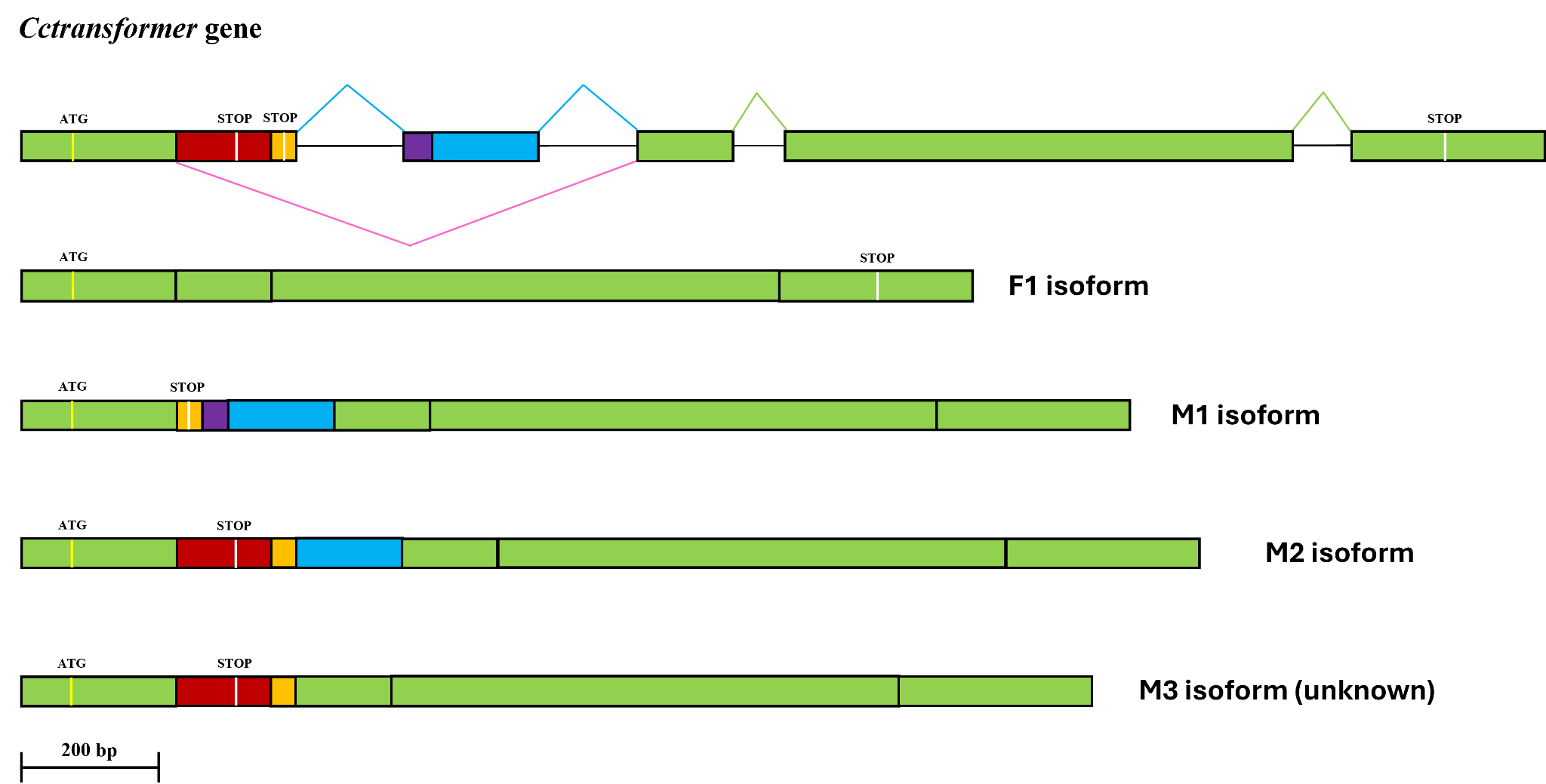


**Fig. S1. Genomic organization and sex-specific alternative splicing of the *Cctra* gene.** Green boxes represent common exons shared by all isoforms, while colored boxes (red, orange, purple, and blue) denote male-specific exons included through alternative splicing. Black lines connecting exons indicate introns. The top schematic shows the complete genomic locus with all potential exons and introns. Below, four transcript isoforms are depicted, with the top representing female-specific splicing (lacking male-specific exons) and the bottom two corresponding to known male-specific isoforms incorporating various male-specific exons. The last isoform (encoding the M3 truncated CcTRA protein) represents a novel splicing event obtained after injection of DsiRNAs.


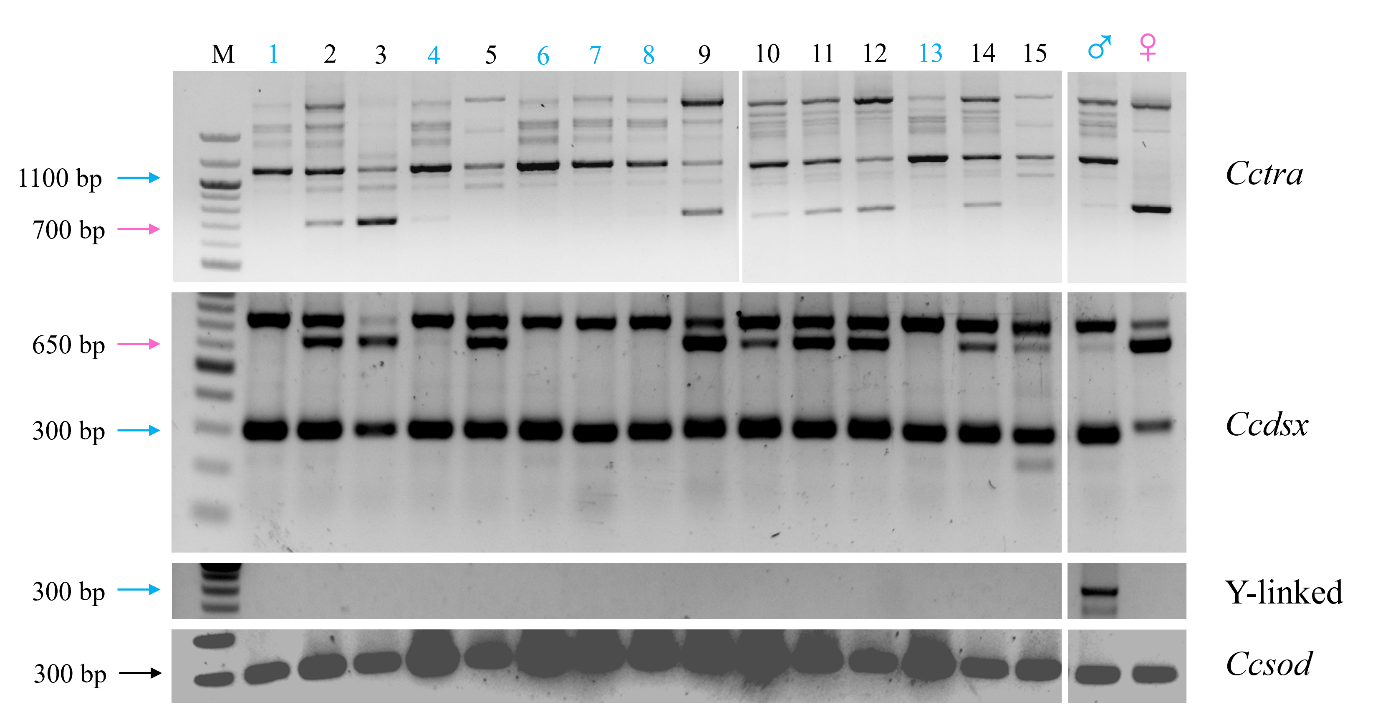


**Fig. S2. RT-PCR of *Cctra* and *Ccdsx* in XX-only adults developed from embryos injection with Cctra #1.** (A) RT-PCR analyses showing the splicing pattern of *Cctra* mRNA: the blue arrow indicates the male-specific splice variant (~1100 bp), and the pink arrow indicates the female-specific variant (~700 bp). (B) RT-PCR analyses showing the splicing pattern of *Ccdsx* mRNA: the blue arrow indicates the male-specific splice variant (~300 bp), and the pink arrow indicates the female-specific variant (~650 bp). (C) RT-PCR analyses showing the presence/absence of a transcript derived from a Y-linked embryonic gene (~300 bp, *MoY*; Meccariello *et al.*, 2019) used as a negative and positive control for the molecular karyotyping. (D) RT-PCR analyses of *Ccsod* mRNA expression (~300 bp) used as a positive control of cDNA and as a negative control for DNA contamination (no intron-containing 400 bp band detected). Blue numbers indicate male individuals confirmed by both phenotypic and molecular analyses; black numbers indicate intersex individuals confirmed by the same criteria. (M): 100 bp DNA Ladder (NEB).

**
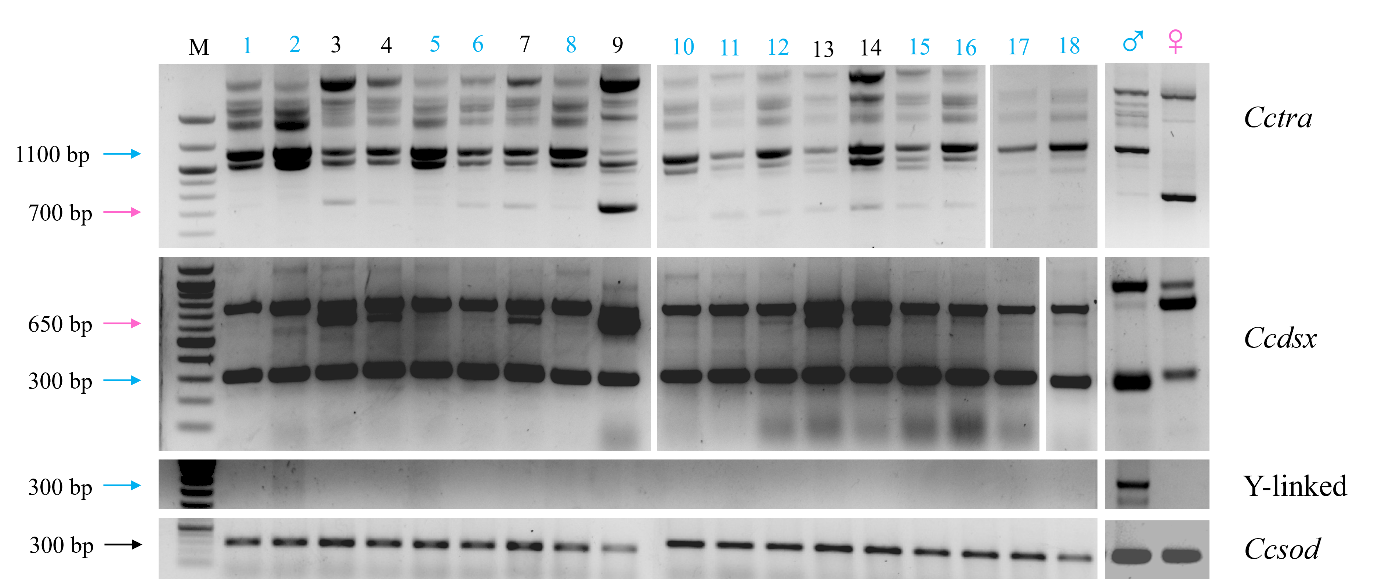
**

**Fig. S3. RT-PCR of *Cctra* and *Ccdsx* in XX-only adults developed from embryos injection with Cctra #2.** (A) RT-PCR analyses showing the splicing pattern of *Cctra* mRNA: the blue arrow indicates the male-specific splice variant (~1100 bp), and the pink arrow indicates the female-specific variant (~700 bp). (B) RT-PCR analyses showing the splicing pattern of *Ccdsx* mRNA: the blue arrow indicates the male-specific splice variant (~300 bp), and the pink arrow indicates the female-specific variant (~650 bp). (C) RT-PCR analyses showing the presence/absence of a transcript derived from a Y-linked embryonic gene (~300 bp, *MoY*; Meccariello *et al.*, 2019) used as a negative and positive control for the molecular karyotyping. (D) RT-PCR analyses of *Ccsod* mRNA expression (~300 bp) used as a positive control of cDNA and as a negative control for DNA contamination (no intron-containing 400 bp band detected). Blue numbers indicate male individuals confirmed by both phenotypic and molecular analyses; black numbers indicate intersex individuals confirmed by the same criteria. (M): 100 bp DNA Ladder (NEB).


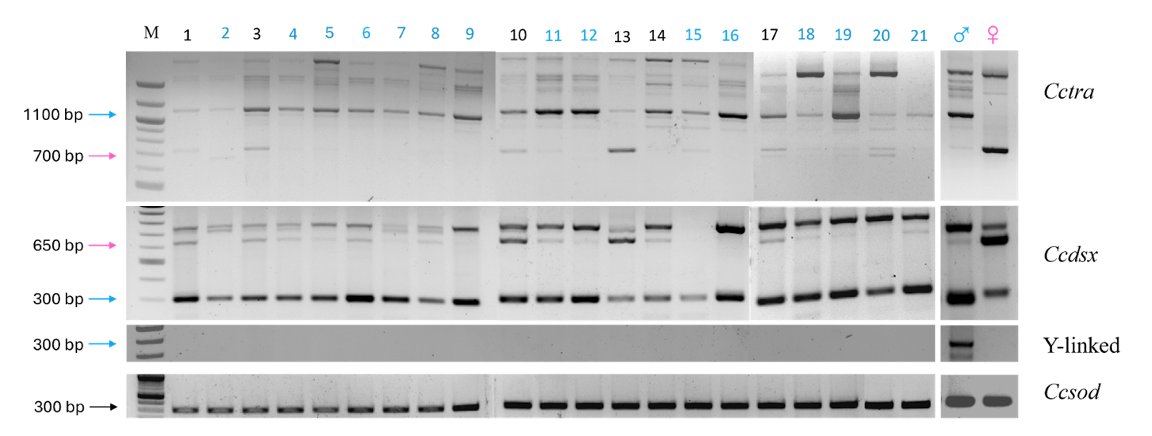


**Fig. S4. RT-PCR of *Cctra* and *Ccdsx* in XX-only adults developed from embryos injection with Cctra #3.** (A) RT-PCR analyses showing the splicing pattern of *Cctra* mRNA: the blue arrow indicates the male-specific splice variant (~1100 bp), and the pink arrow indicates the female-specific variant (~700 bp). (B) RT-PCR analyses showing the splicing pattern of *Ccdsx* mRNA: the blue arrow indicates the male-specific splice variant (~300 bp), and the pink arrow indicates the female-specific variant (~650 bp). (C) RT-PCR analyses showing the presence/absence of a transcript derived from a Y-linked embryonic gene (~300 bp, *MoY*; Meccariello *et al.*, 2019) used as a negative and positive control for the molecular karyotyping. (D) RT-PCR analyses of *Ccsod* mRNA expression (~300 bp) used as a positive control of cDNA and as a negative control for DNA contamination (no intron-containing 400 bp band detected). Blue numbers indicate male individuals confirmed by both phenotypic and molecular analyses; black numbers indicate intersex individuals confirmed by the same criteria. (M): 100 bp DNA Ladder (NEB).

**
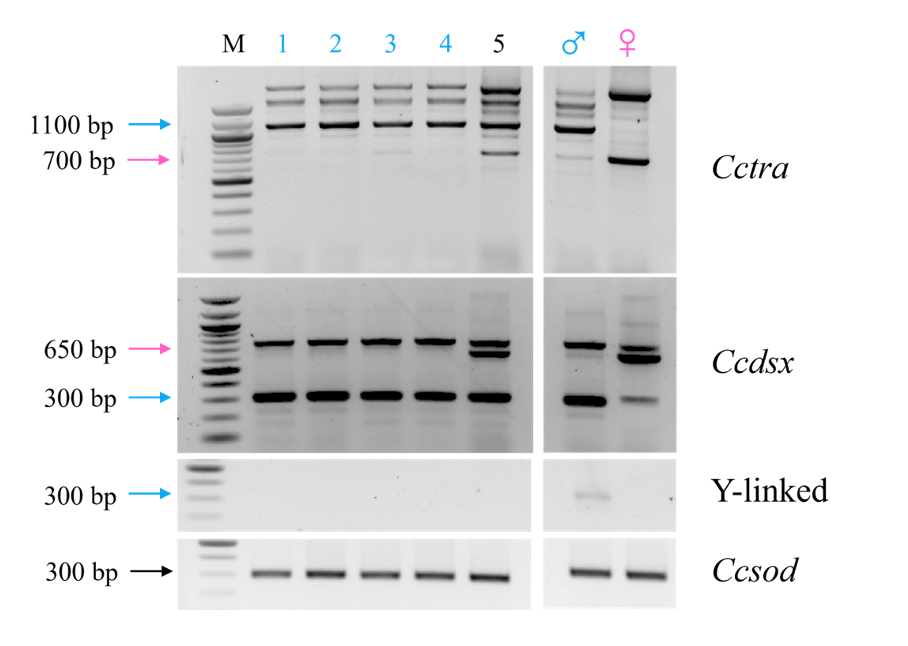
**

**Fig. S5. RT-PCR of *Cctra* and *Ccdsx* in XX-only adults developed from embryos injection with Cctra-2 #1.** (A) RT-PCR analyses showing the splicing pattern of *Cctra* mRNA: the blue arrow indicates the male-specific splice variant (~1100 bp), and the pink arrow indicates the female-specific variant (~700 bp). (B) RT-PCR analyses showing the splicing pattern of *Ccdsx* mRNA: the blue arrow indicates the male-specific splice variant (~300 bp), and the pink arrow indicates the female-specific variant (~650 bp). (C) RT-PCR analyses showing the presence/absence of a transcript derived from a Y-linked embryonic gene (~300 bp, *MoY*; Meccariello *et al.*, 2019) used as a negative and positive control for the molecular karyotyping. (D) RT-PCR analyses of *Ccsod* mRNA expression (~300 bp) used as a positive control of cDNA and as a negative control for DNA contamination (no intron-containing 400 bp band detected). Blue numbers indicate male individuals confirmed by both phenotypic and molecular analyses; black numbers indicate intersex individuals confirmed by the same criteria. (M): 100 bp DNA Ladder (NEB).


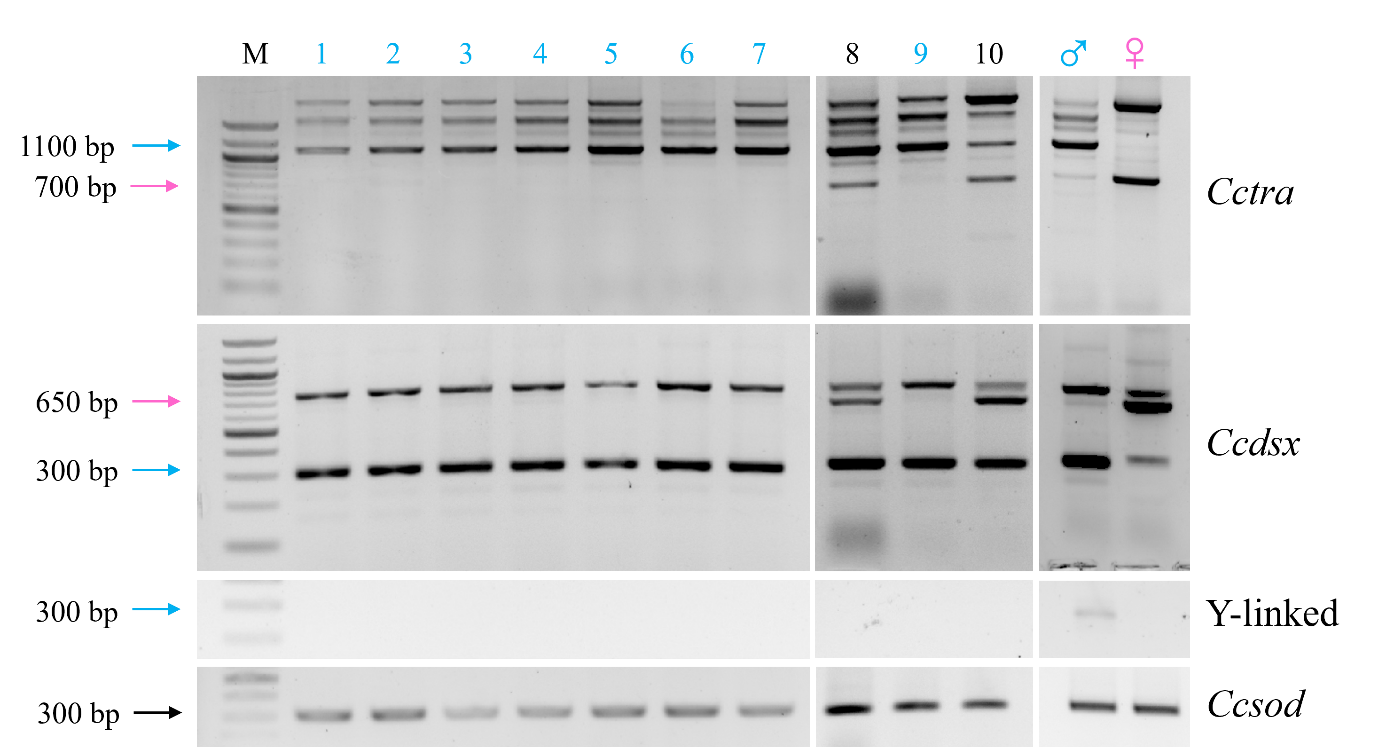


**Fig. S6. RT-PCR of *Cctra* and *Ccdsx* in XX-only adults developed from embryos injection with Cctra-2 #2.** (A) RT-PCR analyses showing the splicing pattern of *Cctra* mRNA: the blue arrow indicates the male-specific splice variant (~1100 bp), and the pink arrow indicates the female-specific variant (~700 bp). (B) RT-PCR analyses showing the splicing pattern of *Ccdsx* mRNA: the blue arrow indicates the male-specific splice variant (~300 bp), and the pink arrow indicates the female-specific variant (~650 bp). (C) RT-PCR analyses showing the presence/absence of a transcript derived from a Y-linked embryonic gene (~300 bp, *MoY*; Meccariello *et al.*, 2019) used as a negative and positive control for the molecular karyotyping. (D) RT-PCR analyses of *Ccsod* mRNA expression (~300 bp) used as a positive control of cDNA and as a negative control for DNA contamination (no intron-containing 400 bp band detected). Blue numbers indicate male individuals confirmed by both phenotypic and molecular analyses; black numbers indicate intersex individuals confirmed by the same criteria. (M): 100 bp DNA Ladder (NEB).
